# Descriptive epidemiology of measles surveillance data, Osun state, Nigeria, 2016–2018

**DOI:** 10.1186/s12889-019-8012-6

**Published:** 2019-12-04

**Authors:** Folajimi O. Shorunke, Oluwatoyin Adeola-Musa, Aisha Usman, Celestine Ameh, Endie Waziri, Stephen A. Adebowale

**Affiliations:** 1 Nigerian Field Epidemiology and Laboratory Training Program, Abuja, Nigeria; 2 Epidemiology Department, Ministry of Health Osun State, Osogbo, Nigeria; 3 African Field Epidemiology Network, Abuja, Nigeria; 4 Department of Epidemiology and Medical Statistics, Faculty of Public Health, University of Ibadan, Ibadan, Nigeria

**Keywords:** Measles surveillance, Seasonal variation, Trend, Osun-state

## Abstract

**Background:**

Globally, an estimate of 254,928 measles cases in 2015 and 89,780 deaths in 2016 occur annually. In Nigeria, measles is the fifth leading causes of under-five child mortality with 342 confirmed cases found in the first 9 epidemic weeks in some states including Osun State. We described the distribution, trend and make projection of measles cases in Osun State.

**Methods:**

The Osun State surveillance weekly reporting data on measles cases from all its 30 Local Government Area (LGA) were reviewed, from January 2016 to December 2018 (*n* = 1205). Data were analyzed using descriptive statistics and a multiplicative time series model (MTSM). The MTSM was used to determine the trend, seasonality in the data and make projections for 2019 and 2020.

**Results:**

Cases of measles were reported across the 30 LGAs of the state between January 2016 and December 2018. The rate of reported cases of measles was 20.2, 34.4 and 28.8 per 100,000 populations in 2016, 2017 and 2018 respectively in Ede south LGA where the highest rates were reported in the 3-year period. Out of the three studied years, year 2017, recorded the highest number of reported cases of measles in Osun State. The trend line for the 3-year period showed a positive correlation (*r* = + 0.4979, *p* = 0.056). The computed quarterly variation for the studied years was 1.094 for the 1st quarter, 1.162 for the 2nd quarter, 0.861 for the 3rd quarter and 0.888 for the 4th quarter. A quarterly projection for 2019 and 2020 showed an increasing trend with the second quarter of each year likely to have the highest reported cases of measles.

**Conclusions:**

Ede south LGA has the highest proportion of reported measles cases in Osun State. Measles cases may increase in years ahead, but the second quarter of a year has the highest number. Government should strengthen the existing framework on measles reduction and more attention should be given to the second quarter of each year.

## Background

Measles disease is caused by a morbilli virus of the paramyxoviridae family and is a contagious respiratory infection. Globally, an estimate of 254,928 measles cases and 89,780 deaths occur annually [1] and the measles case fatality ratios (CFRs) was estimated to be 0.1% [2] in the developed world. Recent estimates of measles CFRs used by WHO in endemic countries range between 0.05–6% [3]. It is a highly infectious disease that will infect about 90% of unvaccinated individuals exposed before the age of 10 [4]. On 16th September 2010, World Health Organization (WHO) countries in the European region came up with a resolution to renew their commitment to eliminate measles and rubella by the year 2015, coming up with several criteria to eliminate the disease in the region, including protection of Europeans against measles and rubella infection [5] which had affected several European countries including Romania and Italy with the largest outbreak reported in southern Italy. Children below the age of 15 had a national incidence of 738/100,000 in 2002 and 544/100,000 in 2003, estimated to more than 100,000 cases within the age group [6]. Despite intensive vaccination activities in the European region, cases were still reported [7].

Africa and Southeast Asia contributed 70% of the 39.9 million global burdens of measles in year 2000 and also 84% of the global measles-related deaths. Only eleven of the African countries including Ethiopia, contributed 66% of this deaths [8]. Sub-Saharan African countries have the highest morbidity and mortality associated with the disease [9]. In this Africa sub-region, the CFR ranges from 3 to 5% but can increase to about 30% during severe outbreaks [9]. In Nigeria, measles exhibits a seasonal pattern with incidence increasing during the dry season, usually between November and May [10] and the disease remains the fifth leading cause of under-five child mortality in the country [11].

The risk of spread and prolonged transmission of measles were associated with the presence of a susceptible population, but vaccination with at least two doses of measles vaccine was the most effective measure for the prevention and control of the disease [7]. Despite the comprehensive campaign and intervention strategies to improve the uptake of vaccination against childhood diseases in Nigeria by Nigerian government and international agencies, the confirmed cases of measles remain high and the case fatality due to the disease is yet to be alleviated [12]. The intervention includes measles-reduction strategy, partnership of other international organizations committed to the support of measles mortality reduction and based on the outcome of this supports, it is still unpleasant that some states in Nigeria continue to face recurrent of epidemics including measles [12]. This may be due to the low coverage in the LGA levels or perhaps because maternal antibodies may have neutralized the vaccine antigen before a specific immune response develops. In Osun State, the childhood diseases vaccination coverage rate is 57.8% which is below the WHO target of 80% and the state is among states with low vaccination coverage in Nigeria [13].

In 2005, Nigeria adopted the program of WHO African region on the reduction of morbidity and mortality associated with measles [9, 10]. Nigeria also adopted the program’s goals revisions and strategies instituted to achieve the goals, this included strengthening infant’s routine measles immunization coverage, provision of a second dose of measles vaccination through supplementary immunization activities (SIA), improving measles case-based surveillance with laboratory confirmation and strengthening the case management [9, 10]. A follow-up campaign has been conducted since the revision of the adopted strategies to increase the coverage of childhood vaccination and immunization in Nigeria. The campaign targets children under-five years between 2008 and 2013. The vaccine of choice is mcv which is administered at 9 months of age, eligible children however get access to more doses through SIA’s conducted one or more times in a year. Findings show that high administrative coverage was observed at the national level but low at the level of Local Government Area. The most recent national survey data on immunization coverage in Nigeria reveals that for children aged 12–13 months, the national coverage for measles was 41.8% (MICS5–2016-2017). Despite these interventions an increase in the cases of measles is still being experiences [10]. In 2008 there were 9510 cases which increased to 17,248 cases by 2011 [14] most of which occurred among children [15]. There were about 194 outbreaks of measles in 2007, 254 in 2008 and 169 in 2009 [16] in addition, 643 outbreaks were confirmed in 83% of the 774 LGAs in Nigeria between epidemic week 1 and 43 of 2013 [17]. The northern part of the country especially Kebbi and Kaduna States had the largest contributions [14].

The WHO IDSR surveillance system and response capacity in any country is important and a lack of adequate capacity can endanger its population. Unfortunately, Nigeria and most developing countries, lack this capacity hence measles outbreaks are not promptly detected, leading to a late or no response hence little public health impact [18, 19].

The objectives of this study are to describe the geographical distribution of measles cases reported in Osun State, determine its trend and make a 2-year projection of the disease in Osun-State. These are with the view to providing situation assessment of the spread of the disease in Osun State and inform policy that will promote reduction in the number of reported cases in the years ahead.

## Methods

### Study design

We conducted a descriptive secondary data analysis of data from Osun State surveillance weekly reporting dataset. The source of the dataset was the Osun State Ministry of Health State Epidemiology department. I received ethics approval from the Osun State Health Research Ethics Committee (OSHREC) to obtain data from the epidemiology department and carry out the analysis as required (Reference number OSHREC/PRS/569 T/155). The dataset presented in excel format was from January 2016 to December 2018. Variables extracted include cases per epidemic weeks, the totals for each year and the various LGAs reporting in Osun State.

### Study setting

Osun State is one of the South-western States in Nigeria, predominated by Yoruba speaking indigenes with a projected population of 4,996,100 in 2018 derived from the 2006 national census. Population of under 5 children is 999,220 and the population of under 15 children is 2,378,144 as at December 2018. There are 832 health facilities in Osun State that conducts routine immunization regularly including some private facilities.

### Sample determination

Measles surveillance system in Osun state uses both active and passive surveillance to achieve a multilevel and multi-directional operation. The surveillance system employs the use of community informants who operate at community level and focal persons who carry out active case search at health facility level. The community informants report to an assigned health facility focal person who has jurisdiction over the community. The Focal Person, in turn reports to the LGA DSNO and complete Case Investigation Form. LGA DSNO reports to the State DSNO and WHO Cluster Consultant. State DSNO reports to Nigeria Centre for Disease Control, then institutes case investigation and reports to the WHO State Office who in turn, reports to the WHO National office.

Suspected cases were determined in the various LGAs through the case definition of measles which is any person with fever and maculopapular (non-vesicular) generalized rash and any one of cough, coryza or conjunctivitis (red eyes) or any person in whom a clinician suspects measles.

A minimum of 5 ml blood samples from suspected cases are collected, triple packaged and sent to the Lagos state teaching hospital for confirmation.

### Data analysis

QGIS 2.18 was used to extract the data features for the 3 years in review and data analyses were done using Microsoft Excel 2016. Charts were used to present the distribution of number and rates of reported cases of measles by LGAs in Osun State. The trends of reported measles cases over the 3-years period were examined using time series analysis. The data were aggregated in 3-months as 1st quarter (January to March), 2nd quarter (April–June), 3rd quarter (July–September), 4th quarter (November–December) to depict the 4-quarters in a year from 2016 to 2018.

The number of cases in a quarter was represented by Y_t_ and thus the trend line (T_t_) was obtained using the model $\frac{y_{t-1}+{y}_t+{y}_{t+1}}{3}$. The plot of the raw data suggests a multiplicative model. Therefore, to obtain the seasonal variation in the data, the multiplicative model was used, and this is mathematically represented by

$$ \mathrm{Seasonal}\ \mathrm{variation}\ \left(\mathrm{SV}\right)=\frac{Y_t}{T_t}\times 100 $$

This seasonality was adjusted by deseasonalize the data to obtain the variation in each quarter of the year as;

$$ \mathrm{Quarterly}\ \mathrm{variation}\ \left({\mathrm{QV}}_{\mathrm{i}}\right)=\frac{\sum_{i=1}^L{SV}_i}{L}-\Delta  SV\left(\frac{\sum_{i=1}^{n-m} SV}{4}\right) $$

Where *∆SV* is the excess of the sum of all the seasonal variation and L is the number of quarters that are presence in the seasonality of a year.

In order to make the projection, the increase in trend line per quarter (ITLPQ) was computed as;

$$ ITLPQ=\frac{T_{t_{Last}}-{T}_{t_{First}}}{n-1} $$

$$ \mathrm{Quarterly}\ \mathrm{projection}=\left({T}_{t_{Last}}+ ITLPQ\right)\times {QV}_i $$

## Results

**Fig. 1:**
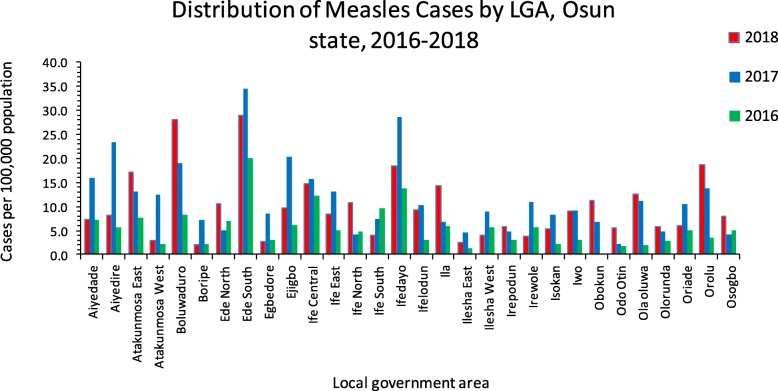
Rates of reported cases of measles per 100,000 populations by year for all LGA in Osun State. Distribution of rates of reported cases of measles per 100,000 populations across the 30 LGA of Osun state Nigeria from January 2016 to December 2018. Each year is represented by the color beside it and the rate of reporting represented by the figures on the Y axis

The total number of suspected cases of measles reported in Osun state between January 2016 and December 2018 were 1205 across the 30 local government area of the state. For the period under review, Ede south LGA had the largest rate of reported measles cases of 20.4 in 2016, 34.4 in 2017 and 28.8 in 2018 per 100,000 populations, followed by Ifedayo, Boluwaduro and Ife central LGA respectively (Fig. 1) see also (Additional file 1). For the entire state, the year 2017, recorded the largest number of reported cases with 512 cases.

**Fig. 2:**
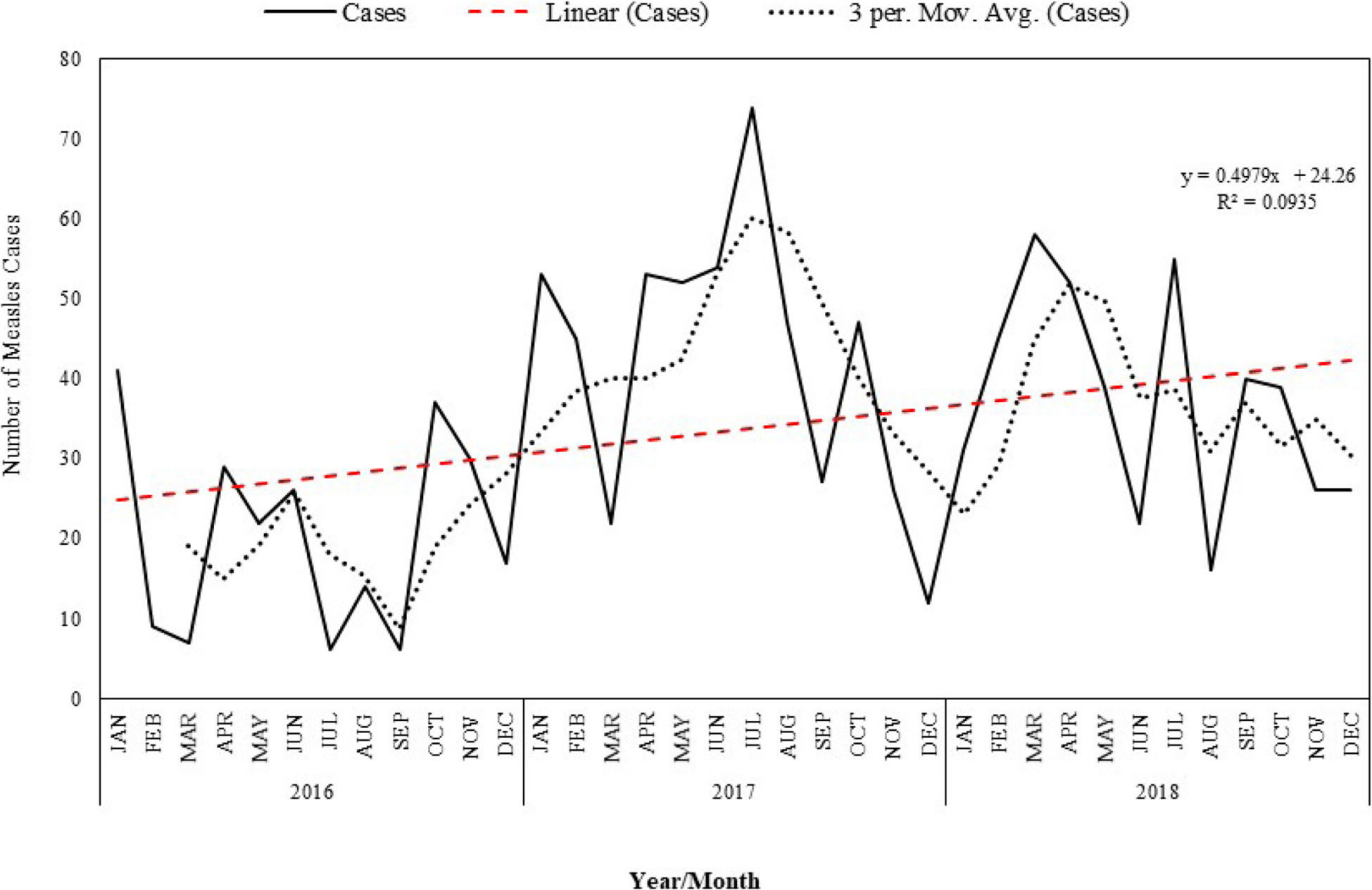
Frequency distribution of observed and 3-months moving average of cases of measles in Osun State. This shows the frequency distribution of the observed cases of measles and the 3 months moving average of the reported cases over the period under review. The thick line represents the cases while the dark broken ken line represent the 3 months moving average. The red broken line is the linear trend line

Figure 2 shows the distribution of cases of measles in Osun State, see also (Additional file 2). The rate of reduction in the reported cases of measles was 0.4979 (*p* = 0.038), an indication that the pattern observed is an increasing trend between January2016 and December 2018. There was a slight variation in the cases of measles in the study area with only 9.4% of the variation being explained by month.

Table 1 shows the process used in the generation of seasonal variation. As observed in the actual data, the seasonal variation was found to be the highest in the second quarter across all the years.

**Table 1:** Estimation of trend and seasonal variation of measles cases in Osun State

	Quarterly observed values	3-months moving total	3-months moving average	Seasonal Variation
2016	57	**–**	**–**	**–**
	77	160	53.33	144.38
	26	187	62.33	41.71
	84	230	76.67	109.57
2017	120	363	121.00	99.17
	159	427	142.33	111.71
	148	392	130.67	113.27
	85	367	122.33	69.48
2018	134	332	110.67	121.08
	113	358	119.33	94.69
	111	315	105.00	105.71
	91	**–**	**–**	**–**

In (Table 2) the data show the adjusted seasonal variation to establish what the exact variation should be for each quarter in the study periods (January–March, April–June, July–September and October–December) of any year. The computed quarterly variation for the studied years was 1.094,1.162,0.861 and 0.888 for the 1st, 2nd, 3rd, and 4th quarter respectively. The increase in trend line per quarter was 5.741.

**Table 2:** De-seasonalization of seasonal variation of measles by quarter in Osun State

	Quarter				
Year	Q_1_	Q_2_	Q_3_	Q_4_	
2016	–	144.38	41.71	109.57	
2017	99.17	111.71	113.27	69.48	
2018	121.08	94.69	105.71	–	
Total	220.26	350.78	260.69	179.05	
Average	110.13	116.93	86.90	89.52	403.48
	0.762	0.762	0.762	0.762	0.762
Quarterly variation	+1.094	+1.162	+ 0.861	+ 0.888	
ITLPQ	5.741				

*ITLPQ* Increase in trend line per quarter

The data as shown in (Table 3) show the projected number of measles cases for the fourth quarter of the year 2018 and all the quarters in years 2019 and 2020. In each of the 2 years where data were projected, the projected cases of measles were highest in the 2nd quarter followed by 1st quarter and 4th and 3rd quarter in that order. In year 2019, the projected cases of measles were 127, 142, 110 and 119 for the 1st, 2nd, 3rd and 4th quarter respectively. This quarterly pattern exhibited in year 2019 was similar to that of year 2020.

**Table 3:** Two-year projection of cases of measles in Osun State

Year	Quarter		ITLPQ	Sum	Quarterly variation	Quarterly Projected figures
2018	Q_1_					
	Q_2_					
	Q_3_	105	5.741			
	Q_4_		5.741	110.741	0.888	98
2019	Q_1_		5.741	116.482	1.094	127
	Q_2_		5.741	122.223	1.162	142
	Q_3_		5.741	127.964	0.861	110
	Q_4_		5.741	133.705	0.888	119
2020	Q_1_		5.741	139.446	1.094	153
	Q_2_		5.741	145.187	1.162	169
	Q_3_		5.741	150.928	0.861	130
	Q_4_		5.741	156.669	0.888	139

**Fig. 3:**
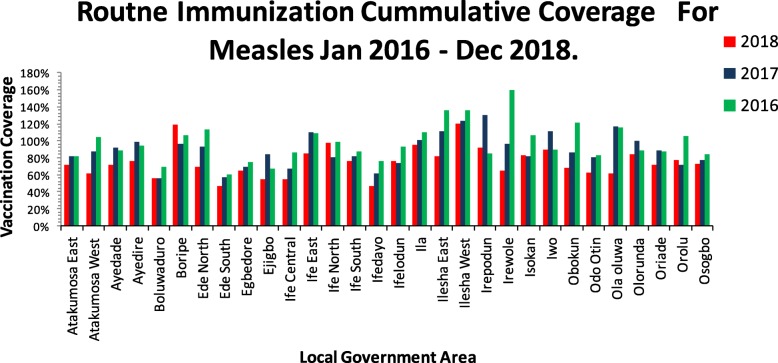
Routine immunization vaccination coverage for measles January 2016 to December 2018. This shows the cumulative measles vaccination coverage for all the LGA in Osun State from January 2016 to December 2018, the vertical axis shows the percentage vaccine coverage while the horizontal axis represent the 30 LGA in Osun state

Figure 3 shows the cumulative measles vaccination coverage for 2016, 2017 and 2018, also see (Additional file 3). In 2016 Ede south LGA had the lowest coverage of 60%, in 2017 Boluwaduro LGA had lowest coverage of 56%, followed by Ede south LGA with a vaccine coverage of 57%, while Ede south and Ifedayo LGA came up with the lowest coverage of 47% in 2018.

## Discussion

The 1205 cases of measles that where investigated across the 30 LGA of Osun State throughout the period under review, showed that measles is endemic in the State. Other related earlier research work has shown a high incidence of measles in the south western region of the country where Osun State is part, with incidences highest in children between one and 5 years of age [10].

Ede South LGA had the highest proportion of reported measles cases within the period under review, this might be as a result of high sensitivity of the measles surveillance system in the local government, or simply because it serves as a link route to and from several other LGAs and other states in the country, this is clearer in the chart of Fig. 1 above, as Ede south measles reporting rates remained high from 2016 to 2018 even when other surrounding LGAs had started experiencing low cases of measles in 2018. The rural LGAs of the state; Boluwaduro, Ila, Ifedayo and others, generally contribute more burden of measles infection than the urban settlements, this could be as a result of lower vaccine coverage in the rural settlements than the urban and also rate of non-compliance tends to be higher in the rural areas probably as a result of illiteracy and poor formal education.

A positive rate of reduction of the distribution of measles cases within the period under review is an indication of an increasing trend, this shows that more cases of measles are being recorded despite all the public health interventions being carried out in the state. The number of reported cases increases from the beginning of the first quarter which is equivalent to the start of the dry season, peaks at the end of the second quarter and then progressively diminishes. This is similar to the pattern reported to be exhibited by measles in Nigeria and WHO Africa region [20]. Increase measles case reporting is also related to the period of school children resumption which is coincidental to the beginning of the dry season and a gradual reduction towards the end of the year when the children are out of school Our projections of 2019 and 2020 cases of measles shows that more cases of measles will be reported in Osun State other things being equal, with the second quarter of the two projected years having the highest expected numbers. These findings are interesting bearing in mind that specific preventive measures, including routine and supplemental immunization, are in place to control the disease [20]. However, the quality of these activities may be doubtful, another explanation for the increase in incidence of measles among vaccinated population could be an infection occurring before development of immunity, a maternal antibody interference or a poor vaccine efficacy due to poor handling and storage [21, 22] a situation of spatial heterogeneity in the populations immune status and the waning of vaccine derived measles immunity with time will bring about a compromised population immunity [23, 24]. The reduction in the level of routine measles vaccination coverage at the state level from 97% in 2016 to 75% in 2018 can also be a possible explanation for the high measles case reporting rate and increase in trend of measles reporting being experienced at the state level, the LGA with the lowest level of vaccine coverage which included Ede south LGA, Ifedayo LGA and Boluwaduro LGA non surprisingly had the largest measles case reporting rate while the LGA with the highest vaccination coverage rates like Ilesha east and Ilesha west LGA has the lowest measles case reporting rates, however a supplementary measles campaign being planned for the second quarter of 2019, if qualitatively implemented, and the coverage is adequate, more rural settlement will be reached and the epidemics will hopefully be milder. With sustenance of proper coverage with routine measles vaccination, the epidemics are expected to stop altogether and transmission will be interrupted. if vaccine coverage is however poor, vaccine immunity will not be sufficient, and susceptible adults will increase. The increasing trend may also be explained by the possibility that reporting might just be getting better as clinics are improving and learning more about reporting. It is then imperative to validate if the observed increase in trend is due to the failure of public health interventions or an improvement in the surveillance system.

Measles pattern in Osun State is endemic but outbreaks occur sporadically peaking in different months during the period under review.

We were limited in our study as we only had access to data from Osun State surveillance weekly reporting with no line list available; hence person characteristic of cases could not be determined. Also the Osun measles case based surveillance system evaluation has not yet been carried out hence we have no insight on the sensitivity of the measles surveillance system and its other system attribute as at the time of this data analysis, but we are in the process of obtaining authority from stake holders to carry out the measles surveillance system evaluation of Osun state measles IDSR reporting system soon.

## Conclusion

This study confirms the endemicity of measles in Osun State. It showed that Ede South LGA has the highest rate of reported measles cases in Osun State. Measles cases may increase in years ahead and the second quarter of a year has the highest number. Low measles vaccination coverage is a likely reason for the increase measles case reporting rate in Ede south LGA.

From our findings, health promotion campaigns through the mass media or via other information and communication materials, such as posters and handbills on the need for measles vaccination and isolation of suspected cases especially during school hours in high reporting areas like Ede south should be instituted by Government. More attention should be given to the second quarter of each year during these campaigns.

## Supplementary information

**Additional file 1.** Rates of reported cases of measles per 100,000 populations from January 2016 to December 2018 for all LGA in Osun State. This file shows the data from which Fig. 1 was derived. The first column for each year (2016–2018) is the LGA the second is the estimated population of each LGA from the Nigeria 2006 national census the third is the number of reported case for that year in each LGA and lastly the computed incidence rate.

**Additional file 2.** Number of reported cases of measles in Osun State by year and months from January 2016 to December 2018. This is the file from which the time trend in Fig. 2 was generated. It consist of the total number of measles cases for each months for all the 3 years under review.

**Additional file 3.** Percentage vaccination rate in Osun State by year, from January 2016 to December 2018. This is the data from which Fig. 3 was derived from. It shows the measles vaccination coverage for the period under review.

## Data Availability

All data generated or analyzed during this study are included in this published article [and its supplementary information files].

## Abbreviations

CFR: Case fatality rate
ITLPQ: increase in trend line per Quarter
LGA: Local government area
OSHREC: Osun State Health Research Ethics Committee
QV: Quarterly variation
SIA: Supplementary immunization activities
SV: Seasonal variation
WHO: World health organization
